# Therapeutic Benefit of Galectin-1: Beyond Membrane Repair, a Multifaceted Approach to LGMD2B

**DOI:** 10.3390/cells10113210

**Published:** 2021-11-17

**Authors:** Mary L. Vallecillo-Zúniga, Peter Daniel Poulson, Jacob S. Luddington, Christian J. Arnold, Matthew Rathgeber, Braden C. Kartchner, Spencer Hayes, Hailie Gill, Jonard C. Valdoz, Jonathan L. Spallino, Seth Garfield, Ethan L. Dodson, Connie M. Arthur, Sean R. Stowell, Pam M. Van Ry

**Affiliations:** 1 Department of Chemistry and Biochemistry, Brigham Young University, Provo, UT 84602, USA; mvallecillo@chem.byu.edu (M.L.V.-Z.); poulson.pd@gmail.com (P.D.P.); jsludd@gmail.com (J.S.L.); cjarnold97@gmail.com (C.J.A.); mattrathgebe13@gmail.com (M.R.); braden.kartchner@gmail.com (B.C.K.); spencerhayes95@gmail.com (S.H.); hailiengill@gmail.com (H.G.); jcvaldoz@up.edu.ph (J.C.V.); jonathan.spallino.9@gmail.com (J.L.S.); seth.garfield18@gmail.com (S.G.); dodson.ethan@gmail.com (E.L.D.); 2 Department of Pathology, Brigham and Women’s Hospital, Harvard Medical School, Boston, MA 02115, USA; cmarthu@emory.edu (C.M.A.); srstowell@bwh.harvard.edu (S.R.S.)

**Keywords:** Galectin-1, LGMD2B, membrane repair, NF-ĸB, inflammation, cytokines, muscular dystrophy

## Abstract

Two of the main pathologies characterizing dysferlinopathies are disrupted muscle membrane repair and chronic inflammation, which lead to symptoms of muscle weakness and wasting. Here, we used recombinant human Galectin-1 (rHsGal-1) as a therapeutic for LGMD2B mouse and human models. Various redox and multimerization states of Gal-1 show that rHsGal-1 is the most effective form in both increasing muscle repair and decreasing inflammation, due to its monomer-dimer equilibrium. Dose-response testing shows an effective 25-fold safety profile between 0.54 and 13.5 mg/kg rHsGal-1 in Bla/J mice. Mice treated weekly with rHsGal-1 showed downregulation of canonical NF-κB inflammation markers, decreased muscle fat deposition, upregulated anti-inflammatory cytokines, increased membrane repair, and increased functional movement compared to non-treated mice. Gal-1 treatment also resulted in a positive self-upregulation loop of increased endogenous Gal-1 expression independent of NF-κB activation. A similar reduction in disease pathologies in patient-derived human cells demonstrates the therapeutic potential of Gal-1 in LGMD2B patients.

## 1. Introduction

Dysferlin is a 230-kDa protein that is highly expressed in skeletal muscle and involved in membrane repair [1]. Membrane repair is essential for maintaining cell integrity and is crucial for stressed skeletal muscle fibers [2,3,4]. Mutated dysferlin leads to compromised membrane integrity and repair, resulting in the phenotypes associated with Miyoshi Myopathy (MM) and Limb-Girdle Muscular Dystrophy 2B (LGMD2B). In addition to diminished membrane repair, muscles in LGMD2B are characterized by wasting, fatty infiltrates (especially in the hip and lower leg), and chronic inflammation [5,6,7,8,9].

Chronic inflammation is responsible for many pathologies seen in LGMD2B [10,11,12]. In particular, the nuclear factor kappa-light-chain-enhancer of activated B cells (NF-κB) signaling complex is highly upregulated in this disease [13]. The NF-κB signal can work through either the canonical or non-canonical pathway. Studies show that Galectin-1 activates the canonical NF-κB pathway in osteoarthritis chondrocytes [14]. The non-canonical NF-κB pathway is associated with the production of pro-inflammatory cytokines in cardiac tissues and the development of tumor necrosis [15,16]. In this study, we show that Gal-1 reduces the expression of the NF-κB proteins in LGMD2B models. Inflammation leads to diminished muscle regeneration, increased production of pro-inflammatory cytokines, and aberrant phagocytic response [11,17,18]. These factors contribute to the muscle wasting, fibrosis, and fatty deposition seen in dysferlinopathies.

Presently, there is neither a cure nor targeted treatment for LGMD2B. Therapies for this disease focus on maintaining muscle strength and ambulation in patients and controlling chronic inflammation [19,20]. Although glucocorticoids have been used as anti-inflammatory treatments in other muscular dystrophies, many do not have the same effect in LGMD2B [21,22]. For example, certain glucocorticoids, such as deflazacort, have detrimental effects on muscle strength and inflammation in LGMD2B [23]. However, there are some steroid treatments showing promise as a therapeutic in animal models of the disease [22]. More viable therapies for LGMD2B, such as protein treatments, are needed.

Gal-1 is a β-galactoside-binding protein that exists in a dynamic equilibrium between monomer and dimer states [24]. This equilibrium, along with the activity of its carbohydrate recognition domain (CRD), contributes to the multifunctional properties of this protein [25,26,27]. Due to a relatively high density of cysteine residues on the surface of the protein, Gal-1 is particularly sensitive to changes in the redox environment [28,29]. The CRD is active when Gal-1 is reduced and inactive when Gal-1 is oxidized [27]. Previous studies show both mouse and human Gal-1 reduced disease pathology in the *mdx* mouse model of Duchenne muscular dystrophy [30]. There were no reports of adverse immunogenicity while using human Gal-1 in the mouse model. Additionally, we did not observe adverse reactions or signs of elevated inflammation in our previous or current study [31]. This is likely because human and mouse Gal-1 sequences are 88% homologous. Although there are some minor structural differences, the CRD residues are 100% conserved between species (https://blast.ncbi.nlm.nih.gov/Blast.cgi (accessed on 10 November 2021)). For these reasons, we used the human form of Gal-1 in our in vitro, ex vivo, and in vivo studies. In this paper, we demonstrate that the reduced dimeric form of Gal-1 is responsible for inducing improvements to muscle membrane repair, while the monomeric form is responsible for changes in inflammation. Here, we not only present in vitro and in vivo results in mice, but also present significant data in patient-derived dysferlin-deficient human myotubes, substantiating our claim that Gal-1 treatment increases muscle repair and decreases markers of inflammation. Previous research shows that both of these processes lead to increased functional activity in dysferlinopathy models.

## 2. Materials and Methods

### 2.1. Galectin-1 Construction, Production, and Purification 

Recombinant Human Galectin-1 (rHsGal-1) was produced and purified as described by Vallecillo-Zúniga et al. [31]. In summary, LGALS1 gblock was cloned into the pET29b(+) vector and transformed into BL21(DE3) competent E. coli cells, grown, and induced with 0.1 mM IPTG. rHsGal-1purification was accomplished as described in Stowell et al. [32]. rHsGal-1 bacterial lysate was passed through a lactoyl-Sepharose column (Sigma-Aldrich, St. Louis, MO, USA). Galectin was eluted with 14 mM β-mercaptoethanol (β-ME) and 100 mM Lactose in 1X PBS. Protein fraction absorbance was read at 280 nm on BioPhotometer (Eppendorf, Hamburg, Germany), and fractions with an absorbance of greater than 0.5 were kept and stored at −80 °C. To activate the CRD, factions were passed over a PD-10 desalting column (GE Health Care, Chicago, IL, USA) and stored at 4 °C.

Monomeric Galectin-1 (mGal-1) was constructed, produced, and generously provided by Dr. S. Stowell at Emory University. This was achieved by capping the N-terminus with Lys, which severely inhibits Gal-1 dimerization [33]. Dimeric Galectin-1 (dGal-1) was constructed using a 2 Gly residue linking the N-terminus of one subunit to the C- of the other. The rHsGal-1GG construct, which contains two rHsGal-1 CRDs, was connected by a glycine–glycine linker that maintains the CRD orientation of wild-type galectin-1. The nucleotide sequences were constructed in SnapGene (GSL Biotech, Chicago, IL). Vector assembly was completed by Twist Bioscience (San Francisco, CA, USA). dGal-1 purification was accomplished as described in Stowell et al. [32]. mGal-1 and dGal-1 forms were either left in a native reduced form or oxidized using 1 μM H_2_O_2_ [27].

### 2.2. Animal Care 

Three- to nine-month-old male Bla/J mice (Dysf^−/−^ (B6.129-Dysf^tm1Kcam^/J)) were provided by the Jain Foundation. Mice were housed in an approved facility at Brigham Young University. All procedures were performed in accordance with Brigham Young University IACUC-approved protocol. Purified rHsGal-1 was used as a treatment in in vivo studies. Protein concentration was analyzed using a Bradford assay protocol following the manufacturer’s instructions. BLA/J mice were weighed and dosed with various doses of rHsGal-1. Treatment was delivered with 1 mL insulin syringes (BD, San Jose, CA, USA Cat #329420) intraperitoneally (IP).

### 2.3. Cell Culture

Immortalized primary H2K A/J^−/−^ (A/J^−/−^) myoblasts were cultured as described in Vallecillo-Zúniga et al. [31]. Mouse myoblasts were plated onto T75 flasks (SARSTED No. 83.3911.302, Newton, NC, USA) or standard 35 mm glass-bottom dishes (Mat Tek, No. 1.5 Coverslip, 14 mm Glass Diameter, Uncoated. Ashland, MA, USA), seeding at a density of 5555 cells/cm^2^ and incubated at 33 °C in 10% CO_2_. At 80–90% confluency, myoblasts were differentiated and treated with or without 0.11 µM rHsGal-1 for 48–72 h at 37 °C and 5% CO_2_. Twitching of myotubes was quantified using ImageJ by selecting 10 different fields of view and calculating the number of visible twitching myotubes over the total number of myotubes present.

Patient-derived dysferlin deficient cells (cell line “814”) were given, courtesy of the Jain Foundation, Dr. Simone Spuler, and Dr. Vincent Mouly. The dysferlin mutation was in Exon 38: c.4022 > TC HMZ, r.4022U > C, p. L1341P (Homozygous). The tissue originated from the vastus lateralis of a 60-year-old of unknown gender [34].

### 2.4. Muscle Fiber Isolation

Muscle fiber isolation was performed as described in Vallecillo- Zúniga et al. [31]. In summary, a 6-well plate (Cat. No. 665 180, Grenier Bio-One) was used as a digestion plate and prepared as described in Demonbreun et al. [35]. When the mice were sacrificed, the flexor digitorum brevis (FDB) was excised. Non-muscle tissues, including tendon, nerve, and overlying fascia, were carefully removed, and muscles were incubated in Collagenase II (2.5 U/mL, ThermoFisher, #17101015) in DMEM for 60–90 min at 37 °C. Next, by using a small-bore pipette, the fibers were transferred to 35 mm glass-bottom microwell dishes (Cat. No. P35GCol-1.0-14-C, MatTek, Ashland, MA, USA) and allowed to attach for at least 15 min.

### 2.5. Laser Injury

Laser injury assay was performed as described in Vallecillo-Zúniga et al. [31]. A/J^−/−^ non-treated or treated myotubes were prepared for laser injury in 35 mm glass-bottom microwell dishes (Cat. No. P35GCol-1.0-14-C, MatTek, Ashland, MA). After washing with PBS, the myotubes were incubated in PBS enriched with 2.5 μM FM™ 1–43 dye (N-(3-Triethylammoniumpropyl)-4-(4-(Dibutylamino) Styryl) Pyridinium Dibromide)3,5 (Thermo Scientific, Cat. No. T35356, Waltham, MA, USA) for 5 min before injury. Digested rHsGal-1 or PBS-treated Bla/J myofibers were placed onto 35 mm glass-bottom microwell dishes (Cat. No. P35GCol-1.0-14-C, MatTek) containing 300 µL of PBS. Myofibers were incubated with 2.5 μM FM™ 1–43 dye (N-(3-Triethylammoniumpropyl)-4-(4-(Dibutylamino) Styryl) Pyridinium Dibromide)3,5 (Thermo Scientific, Cat. No. T35356, Waltham, MA, USA) for 5 min before injury. The kinetics of repair were examined by determining the total change in fluorescence intensity of FM™ 1–43 dye (ΔF/F0, where F0 is the original value at time 0) at the site of the wound for each time point relative to the pre-injury fluorescent intensity that was measured using ImageJ [31]. 

### 2.6. RayBio Mouse Inflammation Array 

Media from NT and rHsGal-1 treated A/J^−/−^ myotubes were collected after 48 h. Cytokine expression was measured using the Mouse Inflammatory Array C1 (RayBiotech, Cat.No.AAM-INF-1-8, Peachtree, GA, USA) according to the manufacturer’s instructions. Membranes were imaged using a FluorChem imaging system (Alpha Innotech, San Jose, CA, USA). The membranes were quantified using ImageJ software as described in Schindelin et al. [36].

### 2.7. Immunofluorescence

A/J^−/−^ myoblasts were cultured onto 35 mm glass-bottom microwell dishes (Cat. No. P35GCol-1.0-14-C, MatTek, Ashland, MA, USA), fixed in 4% paraformaldehyde, permeabilized in 0.1% triton X-100 (in PBS), and blocked using PBST blocking solution for 1 h at room temperature. The myotubes were then incubated overnight at 4 °C with the appropriate primary antibody: NF-κB p65 (Cat. No. 8242s, Cell Signaling, 1:5000), Phalloidin-Alexa Fluor 647 (Cat No. A2287, Invitrogen, Rockford, IL, USA, 1:50), β-Tubulin Loading Control, BT7R, (Cat. No. MA5-16308, ThermoScientific, Waltham, MA USA, 1:2000), GAPDH (Cat. No. MA5-15738, Invitrogen, Rockford, IL, USA, 1:1000), and Anti-β-actin (Cat. No. A5441, Sigma-Aldrich, St. Louis, MO, USA. 1:5000). After washing primary antibodies, blots were probed using the following secondary antibodies: goat anti-mouse IgG (H + L) highly cross-adsorbed secondary antibody, Alexa Fluor Plus 488 (Cat. No. A32723, ThermoFisher, Waltham, MA USA, 10 μg/mL), or Alexa Fluor 555 Goat Anti-Rabbit Secondary Antibody (Cat No. G-21234, 1:1500; Invitrogen, Rockford, IL, USA). Nuclei were counterstained with Hoeschst 33342 (Cat No. 62249, ThermoScientific, 1 μg/mL) or 4′,6-diamindino-2-phenylindole (DAPI) (Cat No. 62248, Thermo Scientific, 1:500). Myotubes were mounted on coverslips using ProLong™ Diamond Antifade Mountant (Cat No. P36965, Invitrogen) and dried overnight. Images were taken on the A TCS SP2 two-photon confocal scanning microscope with LASX imaging software (Leica Microsystems Inc., Buffalo Grove, IL, USA). Fixed Bla/J psoas muscle sections were washed with PBS three times for 5 min each. The sections were then permeabilized in 0.1% Triton X-100 for 15 min and subsequently blocked with EZ-Block PBST (VWR, Cat No. 10005-262) for 1 h at room temperature. Primary antibodies Perilipin-1 (Cat.No.9349, Cell Signaling Technology, Danvers, MA 1:100) and 6x-His Tag Monoclonal Antibody (HIS.H8, Cat. No. 14-6657-80 Invitrogen, Rockford, IL, USA, 1:1000), were diluted in the wash buffer (10% EZBlock in PBST). The slides were left with the primary antibody overnight at 4 °C. The following day, the slides were washed three times with the wash buffer for 5 min, after which the slides were blocked with 3% hydrogen peroxide in methanol. The slides were then washed three times with PBS for 5 min each. The slides were then treated with the HRP conjugated secondary antibody (1:500, Invitrogen, Rockford, IL, USA, Cat. No. G-21234) and left for 1 h at room temperature. The slides were then rinsed once with PBST; after which they were washed with PBST twice PBST for 5 min each. The slides were then washed with PBS once for 5 min and then rinsed once with PBS. DAB (Thermo Scientific, 34002) was prepared according to the manufacturer’s instructions. The slides were treated with DAB for 15 min and counterstained with hematoxylin for 4 min, washed with 100% ethanol, allowed to air dry, and then washed three times with Histo-Clear for 5 min each. Finally, the slides were mounted using Organo/Limonene mounting medium (Sigma-Aldrich, Burlington, MA, O8015) and cured overnight at 37 °C before imaging.

### 2.8. Western Blotting

Whole-cell lysates from A/J^−/−^ myotubes (at 2 to 4 days) and Bla/J gastrocnemius muscles were prepared using RIPA lysis buffer (10 mM Tris-Cl (pH 8.0), 1 mM EDTA, 1% Triton X-100, 0.1% sodium deoxycholate, 0.1% SDS, 140 mM NaCl, and 1 mM PMSF), and Halt™ Protease and Phosphatase Inhibitor Single-Use Cocktail (100X) (Cat No. 78442, ThermoScientific). Protein concentration was determined using the Pierce™ BCA Protein Assay Kit (ThermoScientific). Protein samples were separated under standard conditions on 6–20% SDS-PAGE gels and transferred onto Nitrocellulose Membranes 0.2 μm (Bio-Rad, Cat No.1620150, Hercules, CA, USA) through electro-blotting. After blocking with 5% w/v non-fat dry milk in 1X TBST, membranes were probed overnight for the following mouse, rabbit, or goat monoclonal and polyclonal antibodies: 6x-His Tag Monoclonal Antibody (HIS.H8), (Cat. No. 14-6657-80 Invitrogen, Rockford, IL, USA, 1:1000), Galectin-1 Monoclonal Antibody (6C8.4–1) (Cat. No. 43–7400, Invitrogen, Rockford, IL, USA, 1:1000), TAK-1 Polyclonal Antibody (Cat. No. PA5-17507 Invitrogen, Rockford, IL, USA, 1:1000), NIK Polyclonal antibody (Cat. No. PA5-100732 Invitrogen, Rockford, IL, USA, 1:1000), NF-κB p65 (Cat. No. 8242S Cell Signaling, Boston, MA, USA. 1:1000), Phospho- NF-κB p65 (Cat. No.3033S, Cell Signaling, Danvers, MA, 1:1000), IkBα (Cat. No.4814S, Cell Signaling, 1:1000), IL-4 (Cat. No PA5-25165, Invitrogen, Rockford, IL, USA, 1:1000), TIMP-2 (Cat. No. MA1-774, Invitrogen, Rockford, IL, USA, 1:500), MCP-1 (Cat. No. MA5-17040, Invitrogen, Rockford, IL, USA, 1:500), β-Tubulin Loading Control (Cat. No. 2146S Cell Signaling, 1:1000), GAPDH (Cat. No. MA5-15738, Invitrogen, Rockford, IL, 1:1000), and Anti-β-actin (Cat. No. A5441, Sigma-Aldrich, St. Louis, MO, USA. 1: 5000). After washing primary antibodies, blots were probed using the following secondary antibodies: IRDye^®^ 800CW Donkey Anti-Rabbit IgG (H + L) (Cat No. 926–3221, Licor, Lincoln, NE, 1: 15,000), Goat anti-Mouse IgG (H + L) Highly Cross-Adsorbed Secondary Antibody, Alexa Fluor Plus 800 (Cat No. A-32730, Invitrogen, Rockford, IL, USA, 1: 40,000), Goat anti-Mouse IgG (H + L) Highly Cross-Adsorbed Secondary Antibody, Alexa Fluor 680 (Cat. No. A-21058, Invitrogen, Rockford, IL, USA, 1: 5000), and IRDye^®^ 680RD Donkey Anti-Goat IgG (Cat. No. 926–68074, Licor, 1:5000). The blots were developed using the Odyssey CLx (Model No. 9140, Li-Cor, Lincoln, NE, USA). Quantifications were done by using ImageJ as described in Schindelin et al. [36].

### 2.9. Histology

Bla/J psoas muscles were dissected, embedded in optimum cutting temperature compound (OCT, Cat. No.4583, Sakura, Torrance, CA, USA), and frozen isopentane cooled in a liquid nitrogen bath. Ten micrometers of frozen tissue were cut using a TNF50 Semi-Automatic Cryostat (Tanner Scientific). Sections were placed on 3P white extra slides (Cat. No. 3800200, Leica Microsystems, Buffalo Grove, IL, USA). Sections were processed for immunofluorescence. Digital images were captured with an Olympus microscope (Model BX51).

### 2.10. Statistical Analyses

Data analyses were completed by using Tukey’s multiple comparison test 1-way and 2-way ANOVA and Student’s *t*-test through the GraphPad Prism Software, version 9.0. For membrane repair analysis, the data conferred are the averaged values for all the myotubes used in the analysis and the treatment at individual time points. *p*-values are indicated in the figure when statistical significance is determined for all groups as described in the figure legends.

## 3. Results

### 3.1. Reduced Dimeric Galectin-1 Is Responsible for Optimal Membrane Repair

We designed several synthetic forms of Gal-1 to determine the effect of dimerization and redox states on membrane repair in models of dysferlinopathy (Figure S1). These forms include wild type Gal-1 (WTGal-1), transiently reduced recombinant Human Gal-1 (rHsGal-1), fixed monomeric Gal-1 (mGal-1), and fixed dimeric Gal-1 (dGal-1). WTGal-1 and rHsGal-1 are structurally identical except for the addition of a 6X His-tag on the C-terminus of rHsGal-1 and are comprised of the human LGALS1 gene. For mGal-1, an induced V5D mutation prevents dimerization, as shown by Cho et al. [33]. We formed dGal-1 by fusing two human Gal-1 constructs with a flexible Gly-Gly linker, as described in Earl et al. [37].

**Figure 1:**
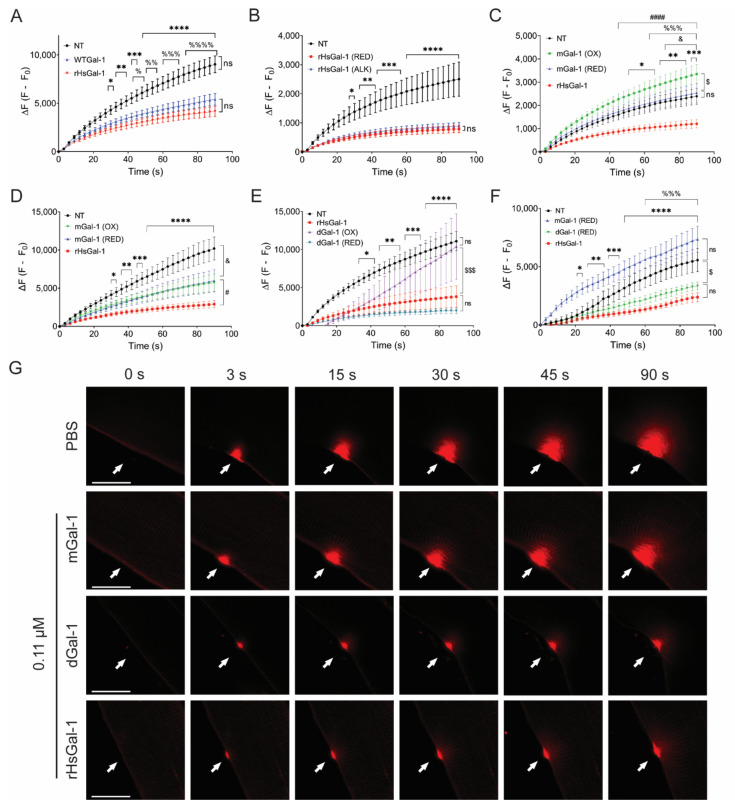
rHsGal-1 is the most efficient type of Gal-1 in helping improve sarcolemmal repair in A/J myotubes and Bla/J myofibers. (**A**) Quantification of the change in fluorescent intensity inside A/J^−/−^myotubes following laser injury when treated with 0.11 μM WT Gal-1 and 0.11 μM rHsGal-1 for 48 h compared to NT A/J^−/−^ myotubes. *, = NT vs. 0.11 μM rHsGal-1. % = NT vs. 0.11 μM WT Gal-1. (**B**) Change in the fluorescent intensity in A/J^−/−^ myotubes following laser injury when treated with 0.11 μM rHsGal-1 and 0.11 μM alkylated rHsGal-1 compared to NT A/J^−/−^ myotubes. (**C**) Quantified laser injury assay displaying membrane repair differences between 10 min treatment of A/J^−/−^ myotubes with 0.11 μM mGal-1 (oxidized), 0.11 μM mGal-1 (reduced), and 0.11 μM rHsGal-1 compared to NT. $, = mGal-1 (oxidized) vs. mGal-1 (reduced), % = mGal-1 (reduced) vs. rHsGal-1, # = mGal-1 (oxidized) vs. rHsGal-1, & = NT vs. mGal-1 (oxidized), * = NT vs. rHsGal-1. (**D**) Quantified laser injury assay displaying membrane repair differences between 48 h treatment of A/J^−/−^ myotubes with 0.11 μM mGal-1 (oxidized), 0.11 μM mGal-1 (reduced), and 0.11 μM rHsGal-1 compared to NT. # = mGal-1 (oxidized and reduced) vs. rHsGal-1, & = NT vs. mGal-1 (oxidized and reduced), * = NT vs. rHsGal-1. (**E**) Quantified laser injury assay displaying membrane repair differences between 48 h treatment of A/J^−/−^ myotubes with 0.11 μM dGal-1 (oxidized), 0.11 μM dGal-1 (reduced), and 0.11 μM rHsGal-1 compared to NT. $ = dGal-1 (oxidized) vs. rHsGal-1, * = NT vs. rHsGal-1 and NT vs. dGal-1 (reduced) and dGal-1 (oxidized) vs. dGal-1 (reduced). (**F**) Quantified laser injury assay displaying membrane repair differences between 2 h treatment of isolated Bla/J mouse myofibers with 0.11 μM mGal-1 (reduced), 0.11 μM dGal-1 (reduced), and 0.11 μM rHsGal-1 compared to PBS. $ = PBS vs. dGal-1 (reduced), % = PBS vs. rHsGal-1, * = rHsGal-1 and dGal-1 (reduced) vs. mGal-1 (reduced). (**G**) Representative images of treated explant Bla/J myofibers during laser injury assay taken at 0 s, 3 s, 15 s, 30 s, 45 s, and 90 s with arrows indicating location of injury. All *p*-values were calculated by Tukey’s multiple comparison test and indicated by ****, %%%%, and #### = *p* < 0.0001; ***, %%%, and ### = *p* < 0.001; **, %%, and ## = *p* < 0.01; and *, %, &, and # = *p* < 0.05. Scale bars = 20 μm. Error bars represent SEM. n ≥ 23 from 2 independent experiments for each group.

Previous work from our lab showed that rHsGal-1 with a 6X his-tag diluted in PBS at a pH of 7.4 beneficially increased membrane repair in dysferlin-deficient myotubes and explants. However, we did not evaluate the effects such as the 6X His tag, alkylation, dimerization, and redox state [31]. Stowell et al. showed that alkylation of Gal-1 provides permanent protein stability through the prevention of cysteine residue oxidation, thus stabilizing the CRD of Gal-1 [28,31]. β-ME is a well-known temporary reducing agent that helps stabilize the protein of interest by cleaving the disulfide bonds between cysteine residues [38]. As explained in the methods section, we purified transiently reduced rHsGal-1 (under a β-ME environment) to ensure protein stability. Prior to the experimental use of rHsGal-1, β-ME was removed by passing the protein through a PD-10 column. To test the activity of rHsGal-1 and WT Gal-1 in membrane repair, A/J^−/−^ myotubes were treated for 48 h with these two forms of Gal-1. After injury, no significant differences were observed in fluorescent intensity between rHsGal-1 and WT Gal-1 treated myotubes (Figure 1A).

We next sought to compare the effects of permanently reduced rHsGal-1 (alkylated rHsGal-1) and rHsGal-1 on membrane repair. After 48 h treatment, myotubes were subjected to a laser injury. The change in fluorescent intensity was nearly identical between the alkylated rHsGal-1 and rHsGal-1 (Figure 1B). Additionally, we found that the final fluorescence intensity between rHsGal-1 and WTGal-1 was 54% and 40% lower when compared to non-treated myotubes, but not statistically significant between the two forms. These results indicate no deleterious effects from the His-tag or alkylation.

The known interactions of Gal-1 are irrevocably connected to the oxidation state. Thus, identifying the relationship between membrane repair and oxidation state is vital to therapeutic development. Previously, our lab showed that a 10 min treatment with rHsGal-1 markedly improved membrane repair [31]. To test the relationship between oxidative state of mGal-1 and repair, we treated A/J^−/−^ myotubes for 10 min with oxidized or reduced mGal-1, using rHsGal-1 as a positive control (Figure 1C). We saw no improvement in myotubes treated with reduced mGal-1, but we observed an increased dye entry of 40% in myotubes treated with oxidized mGal-1 compared to NT myotubes, indicative of decreased repair even when compared to NT A/J^−/−^ myotubes. We then treated A/J^−/−^ myotubes for 48 h with either reduced mGal-1, oxidized mGal-1, or rHsGal-1 (Figure 1D). After 48 h, we saw that both forms of mGal-1 decreased dye entry by 41% compared to NT myotubes, while rHsGal-1 treated myotubes decreased by 72%. The dichotomy of results from the 10 min versus the 48 h treatments with mGal-1 suggests that a longer treatment is necessary for membrane repair when using this form of Gal-1. This implies that signaling pathways must be activated for either redox state of mGal-1 to have a therapeutic effect. This is consistent with reports that monomeric Gal-1 is primarily involved in intracellular signaling [37,39].

To determine whether dGal-1 had similar limitations, we treated A/J^−/−^ myotubes with both oxidized and reduced dGal-1 for 48 h and performed the laser injury assay (Figure 1E). Compared to NT myotubes, oxidized dGal-1 treatment did not show any improvement in membrane repair. However, reduced dGal-1 decreased dye entry by 82%, which is slightly better than the 66% decreased dye entry shown by rHsGal-1. Next, the reduced forms of both mGal-1 and dGal-1 were tested on myofiber explants from Bla/J mice (Figure 1F,G). After a 2 h ex vivo treatment with the various forms of Gal-1, we found that dGal-1 and rHsGal-1 decreased the final fluorescence by 40% and 57%, respectively (Figure 1F,G). Reduced mGal-1 did not decrease fluorescence compared to PBS-treated myofibrils. These data suggest that the dimeric form of Gal-1, as either fixed dGal-1 or rHsGal-1 in monomer-dimer equilibrium, is required to improve membrane repair. 

### 3.2. rHsGal-1 Lowers Expression of Proteins in the NF-κB Pathway

**Figure 2:**
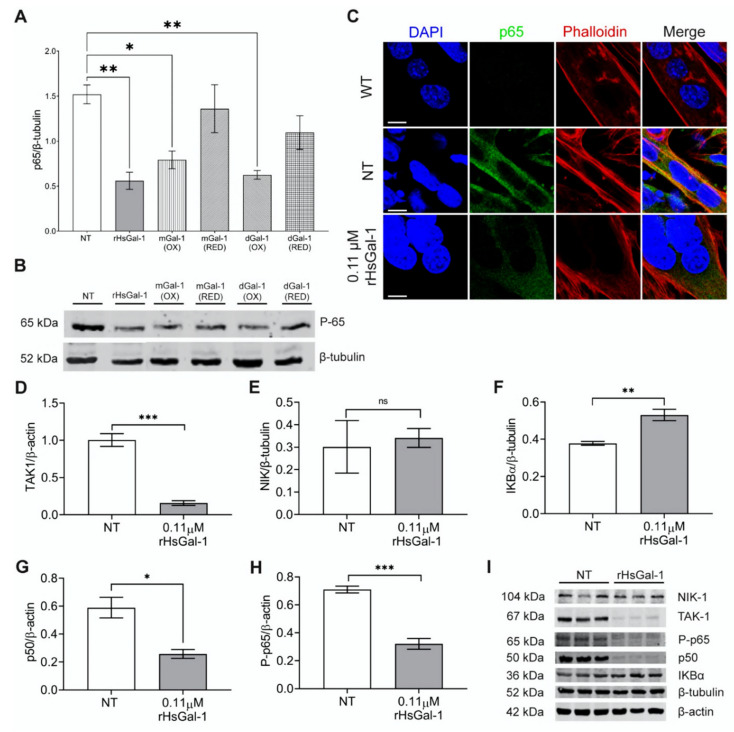
In vitro treatment with rHsGal-1 modulates inflammatory response through the NF-κB pathway. (**A**) Quantification of expression levels of p65 (normalized to β-tubulin) in 48 h A/J^−/−^ NT or 0.11 μM rHsGal-1 treated myotubes. (**B**) Western blot images showing the p65 expression in NT or 0.11 μM rHsGal-1 48 h A/J^−/−^ treated myotubes. (**C**) Representative images of WT and NT or 0.11 μM rHsGal-1 48 h A/J^−/−^ treated myotubes cultured and immunostained with p65 (green), Phalloidin (red), and DAPI (blue). (**D**–**H**) Western blot quantification of 48 h NT or 0.11 μM rHsGal-1treated myotubes expressing levels of TAK1 (**D**), NIK (**E**), IKBα (**F**), p50 (**G**), and P-p65 (**H**). (**I**) Western blot images of 48 h NT or 0.11 μM rHsGal-1treated myotubes expressing NF-κB inflammatory subunits quantified in D-H. n = 3 in each group. A. * *p* < 0.05 and ** *p* < 0.01 NT vs. all forms of Gal-1. D-H. * *p* < 0.05, ** *p* < 0.01, *** *p* < 0.001 NT vs. rHsGal-1. Data are represented as SEM.

Chronic inflammation is a common disease pathology of LGMD2B and a desirable therapeutic target. We hypothesized that Gal-1 would reduce certain NF-κB markers that are upregulated in LGMD2B. We first examined the ability of our various recombinant forms of Gal-1 to modulate the activation of the NF-κB pathway. A/J^−/−^ myotubes were treated for 48 h with oxidized and reduced forms of Gal-1, and lysates were probed for p65. We found that only rHsGal-1, oxidized mGal-1, and oxidized dGal-1 reduced p65 levels (Figure 2A,B). These results were confirmed via immunofluorescent imaging of p65 in A/J^−/−^ myotubes (Figure 2C). Based on the fact that only rHsGal-1 produced optimal effects in both the injury repair assay and NF-κB pathway activation, we used this form in the rest of our experiments.

There are two activation pathways of NF-κB inflammation: canonical activation via TAK1 and non-canonical activation via NIK. It is unknown how Gal-1 regulates the non-canonical NF-κB pathway. As such, we wanted to determine which inflammatory pathway Gal-1 regulates in LGMD2B models. We achieved this by probing for the respective proteins of each pathway. The rHsGal-1 treatment lowered levels of TAK1 by 84%, but did not induce changes in NIK, indicating that the reduction in inflammation is due to modulation of the canonical NF-κB pathway (Figure 2D,E). We further examined multiple proteins downstream of TAK1, including IKBα, p50, and phospho-p65 (P-p65) (Figure 2F–I). These results show a dramatic decrease in inflammatory transcription factors p50 and P-p65, by 56% and 55%, respectively, as well as an increase in the inhibitory protein IKBα by 40%. To verify the presence of P-p65, we visualized its location using immunofluorescent microscopy. We saw a decreased expression of P-p65 in A/J^−/−^ myotubes when treated with rHsGal-1 (Supplementary Figure S2). This overall reduction in inflammatory markers indicates that the monomeric rHsGal-1 is largely responsible for the likely decrease of NF-κB inflammation in cellular models of LGMD2B.

### 3.3. The Therapeutic Window for In Vivo rHsGal-1 Is from 1.35 to 8.1 mg/kg

**Figure 3:**
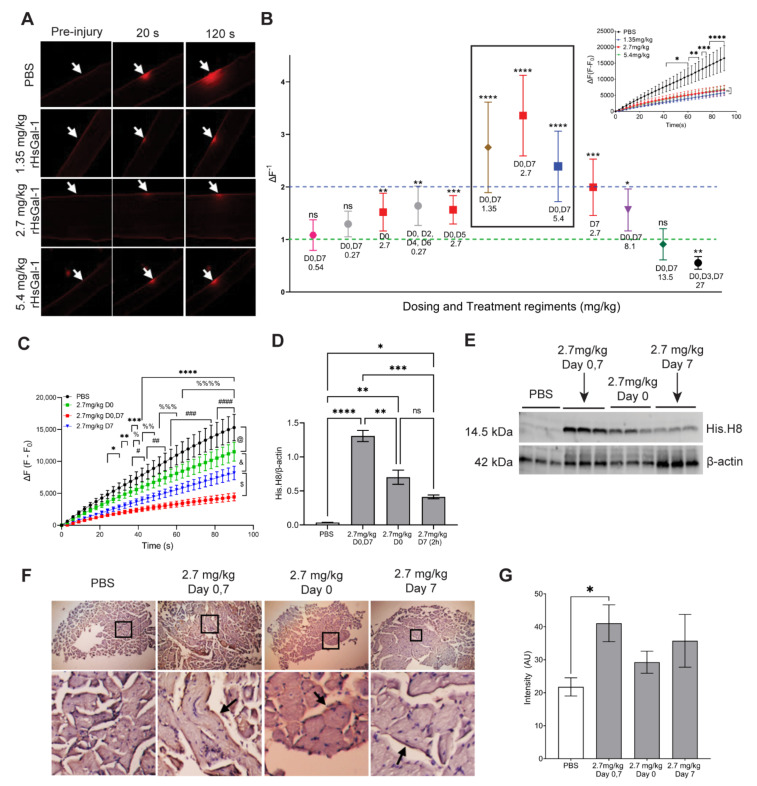
The best dose to improve membrane repair in vivo was 2.7 mg/kg rHsGal-1. (**A**) Representative images from laser injury assay on myofibers taken from Bla/J mice PBS or rHsGal-1treated in vivo. (**B**) Average end fluorescence change from several dosages and regiments of rHsGal-1. Points above red line indicate increased membrane repair from control and points below indicate decreased membrane repair from control. Inset shows representative graph of treatments with fold change > 2. (**C**) Laser injury quantification of D0, D7; D0; and D7 treatments of rHsGal-1. * = D0, D7 vs. PBS; % = D7 vs. PBS; # = D0 vs. PBS. (**D**) Quantification of His.H8 western blot. (**E**) Western blot for His.H8 from gastrocnemius extracted from mice treated PBS or rHsGal-1 for various treatment schedules. (**F**) Representative images of H-DAB staining for rHsGal-1 from specified treatment groups. (**G**) Quantification of H-DAB intensity for rHsGal-1 in psoas. Values were measured by Tukey’s multiple comparison test and indicated by: ****, %%%% = *p* < 0.0001; ***, %%%, and ### = *p* < 0.001; **, %%, and ## = *p* < 0.01, and *, $,#, &, and @ = *p* < 0.05 between control and rHsGal-1 treated mice.

A broad therapeutic window is a desired trait for drug therapies. Based on our in vitro dosage and the dosages used in the mdx DMD model, we predicted the therapeutic dosage for our in vivo model to be 2.7 mg/kg [30,31]. Our previous work showed that an ex vivo treatment of myofibers taken from SJL/J (Dysf^−/−^) and Bla/J mice 2 h prior to injury resulted in an increased membrane repair capacity [31]. Here, we used ex vivo membrane repair assays to determine the optimal rHsGal-1 dose and to define the in vivo therapeutic window in LGMD2B murine models (Figure 3A,B, Figure S3, and Figure S4, and Table 1). Intraperitoneal injections of Gal-1 have been successfully utilized in other dystrophic mouse models [30]. Wuebbles et al. showed that intravenous (IV) rHsGal-1 doses greater than 2.5 mg/kg were lethal due to the ability of Gal-1 to induce hemostasis and thrombosis [40]. Thus, all doses were given intraperitoneally (IP). Day 7 (D7) doses were given 2 h prior to sacrificing the mouse, based on previous work in the Bla/J mouse model [31]. The change in fluorescent intensity for each rHsGal-1 treatment was normalized to PBS-treated groups to allow comparisons between experiments (Figure 3B, Figure S3, and Figure S4). We found that most doses of rHsGal-1 either positively improved membrane repair or had no detrimental effect. Only 27 mg/kg rHsGal-1 given IP three times a week proved detrimental to membrane repair, although we observed no other signs of animal distress or toxicity. Three doses, 1.35 mg/kg, 2.7 mg/kg, and 5.4 mg/kg, given at Days 0 (D0) and 7, proved to have “clinically significant” impacts on membrane repair (greater than 2-fold change from PBS-treated mice) (Figure 3B, inset).

Since 9 out of the 12 dosing schemes included a Day 7 treatment, we evaluated whether the rHsGal-1 dose provided 2 h prior to sacrifice was necessary to gain maximum improvement in membrane repair. Mice were treated on either Day 0 only, Day 7 only, or Days 0 and 7. Laser injury assay showed that the combined Days 0 and 7 treatments improved membrane repair the most (71% decrease in final fluorescence intensity). The individual Day 0 and Day 7 treatments also showed significant improvements to membrane repair (final fluorescence intensity decreased 25% and 47%, respectively). This suggests that rHsGal-1 provides immediate and cumulative benefits to membrane repair (Figure 3).

To assess the relationship of treatment schedule to the amount of rHsGal-1 in tissues, we used a western blot analysis to probe for the His-tag present in our rHsGal-1. We found that tissues from mice treated on Days 0 and 7 had 87% more rHsGal-1 than mice treated on Day 0 and 220% more rHsGal-1 than mice treated on Day 7 (Figure 3D,E). Oxidized 3,3′-Diaminobenzidine (H-DAB) staining showed that only tissues from Bla/J mice treated with rHsGal-1 on Days 0 and 7 had significantly more rHsGal-1 in the tissues than non-treated mice (Figure 3F,G). These results show that rHsGal-1 has benefits at both 2 h and week-long treatments and has additive effects on membrane repair with both treatments.

### 3.4. One-Month rHsGal-1 Treatment Improves Translational and Biochemical Measures of LGMD2B

**Figure 4:**
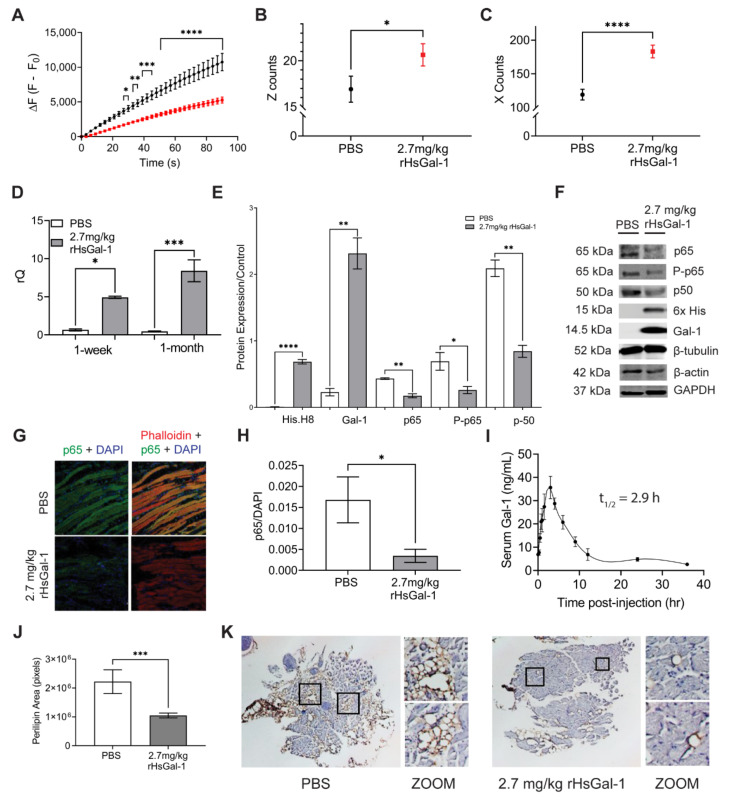
rHsGal-1 improves membrane repair and exploratory activity and decreases inflammatory markers in Bla/J mice after one-month treatment. (**A**) Quantification of laser injury on muscle from BLA/J mice treated for one month with either 2.7 mg/kg rHsGal-1 or PBS. (**B**) Average number of rearing events during first hour placed in CLAMS cages for 2.7 mg/kg rHsGal-1 treated, and PBS treated BLA/J mice. (**C**) Average number of times the mice crossed the x-axis during first hour placed in CLAMS cages for 2.7 mg/kg rHsGal-1 treated and PBS treated control BLA/J mice. (**D**) RT-qPCR results for Gal-1 gene expression in the psoas of Bla/J mice treated for 1 week or 1 month with PBS (control) or 2.7 mg/kg rHsGal-1. (**E**) Quantification of Western blot comparing levels of Gal-1, His-Tag, p65, P-p65, p50, all normalized to β-tubulin control. Tissue was taken from Bla/J mice treated with 2.7 mg/kg rHsGal-1 or PBS (control) for one month. (**F**) Western blot images from homogenized muscle tissue from PBS or 2.7 mg/kg rHsGal-1 treated BLA/J mice. (**G**) Representative images of immunofluorescence on Bla/J mice treated for one month with 2.7 mg/kg rHsGal-1 or PBS (control). Samples were stained with p65, DAPI, and Phalloidin. (**H**) Quantification of immunofluorescence of p65 normalized to DAPI control on mouse psoas muscles either treated with 2.7 mg/kg rHsGal-1 or PBS. (**I**) Concentration of Gal-1 present in serum of Bla/J mice treated with rHsGal-1 from time of treatment (t = 0) to 12 h after treatment. (**J**) Quantification of perilipin stain in mouse psoas muscles treated with PBS or 2.7 mg/kg rHsGal-1 for one month. (**K)** Representative images of histology performed on BLA/J mouse muscles treated with either PBS or rHsGal-1. Mouse psoas muscles were sectioned and immunostained with perilipin (DAB) and counterstained with hematoxylin. Values for all graphs were measured by Tukey’s multiple comparison test and indicated by: **** *p* < 0.0001, *** *p* < 0.001, ** *p* < 0.01, and * *p* < 0.05 between control and rHsGal-1 treated mice.

After determining that a 2x/week treatment of 2.7 mg/kg rHsGal-1 improved membrane repair, we tested its efficacy during a one-month study. Nine-month-old Bla/J mice were treated weekly with 2.7 mg/kg/wk rHsGal-1 for four weeks. At the end of the four-week study, we assessed membrane repair capacity, functional activity, histopathology, and inflammation. Using activity monitoring cages, we saw significant increases in rearing events (Z counts) and horizontal movement (X counts) after the one-month treatment with rHsGal-1 (1.22 and 1.54-fold difference, respectively; Figure 4B,C). Additionally, we found that membrane repair was improved with a final fluorescence decrease of 51% compared to PBS treated Bla/J mice (Figure 4A).

Studies in *mdx* mice and our previous study of A/J^−/−^ myotubes showed that rHsGal-1 treatment resulted in a positive feedback loop resulting in upregulating endogenous Gal-1 [31]. To determine if this apparent positive feedback loop existed in vivo, we used RT-qPCR to determine Gal-1 transcription levels in the psoas, the most affected muscle in the Bla/J mouse model [41]. We found a 7.5 and 18-fold increase in LGALS1 after 1-week (D0, D7 regiment) and 1-month treatments, respectively (Figure 4D). This data provided evidence of a positive feedback loop, where exogenous Gal-1 induces expression of endogenous Gal-1. An ELISA assay was used to measure the concentration of Gal-1 in serum. Results showed that there was a baseline concentration of Gal-1 of 6.9 ng/mL. A one-time IP treatment with 2.7 mg/kg revealed that the serum Gal-1 levels reached peak concentration at approximately 3 h and by 12 h had returned to baseline concentration. The half-life of rHsGal-1 treatment in the blood of Bla/J mice is 2.9 h (Figure 4I).

In order to determine if the reduction in inflammatory markers seen in vitro was recapitulated in vivo, we probed for p65 in the psoas of mice treated with rHsGal-1 and PBS for one month. Immunofluorescence imaging revealed a reduction in the normalized area of p65 (Figure 4G,H). This was confirmed via western blot of the gastrocnemius muscle (Figure 4E,F). Additionally, the expression of other NF-κB proteins, p50 and phospho-p65, was significantly reduced.

Patient and animal model histopathology shows upregulation of fibroadipogenic progenitors in slow twitch myofibers, which correlates with disease pathophysiology [42,43,44]. To investigate the effect of rHsGal-1 on this aspect of the disease, we probed psoas tissue sections for perilipin, a marker of lipid infiltrate. We saw that the one-month treatment reduces perilipin area by 50% (Figure 4J,K). Additionally, we observed that after 15 days, A/J^−/−^ myotubes treated with rHsGal-1 showed significantly more twitching ability compared to NT myotubes (Figure S5 and Supplementary Video S1). Together, these results show that treatment with rHsGal-1 improves muscle function (activity), muscle membrane repair, pathology, and inflammation.

### 3.5. rHsGal-1 Treatment Upregulates Anti-Inflammatory Cytokine Secretion In Vitro and In Vivo

**Figure 5:**
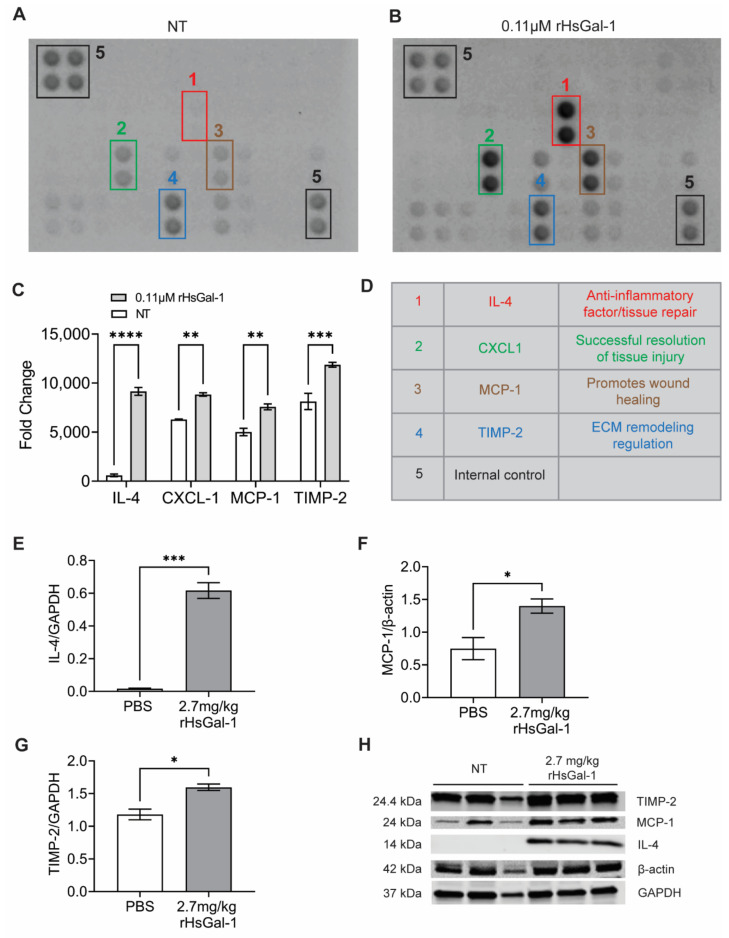
rHsGal-1 treatment upregulates anti-inflammatory cytokines. (**A**) Cytokine array expression from NT A/J^−/−^ myotubes cultured in differentiation media for 48 h. (**B**) Cytokine array expression from A/J^−/−^ myotubes after 48 h in differentiation media supplemented with 0.11 μM rHsGal-1. (**C**) Mean pixel density of relative cytokine expression from A and B. (**D**) Schematic reference of the significantly upregulated cytokines after 48 h treatment with 0.11 μM rHsGal-1. (**E**) Quantification of Western blot probing for IL-4. (**F**) Quantification of Western blot of MCP-1 levels normalized to β-actin in cells treated with PBS (control) or 2.7 mg/kg rHsGal-1. (**G**) Quantification of Western blot of TIMP-2 normalized to GAPDH levels in cells treated with PBS (control) or 2.7 mg/kg rHsGal-1. Tissues taken from mice treated for 1 month. (**H**) Images of Western blot for TIMP-2, MCP-1, β-actin, and GAPDH on tissues from Bla/J mice treated with PBS (control) or 2.7 mg/kg rHsGal-1 for one month. n = 4 for each group. * *p* < 0.05, ** *p* < 0.01, *** *p* < 0.001, **** *p* < 0.0001 NT vs. rHsGal-1. Bars are represented as SEM.

To further investigate the effect of rHsGal-1 treatment on the NF-κB pathway, we quantified changes in secreted cytokines in dysferlin models with treatment. We tested cell culture media of A/J^−/−^ myotubes NT or treated with 0.11 μM rHsGal-1 using a mouse cytokine profiler (Figure 5A,B). This assay revealed that IL-4, CXCL-1, MCP-1, and TIMP-2 cytokines were upregulated during 48 h rHsGal-1 treatment by 15.5, 1.4, 1.5, and 1.5-fold, respectively (Figure 5C). Although there are several functions for each cytokine, the commonality between them is that each has either anti-inflammatory properties or promotes tissue remodeling and regeneration (Figure 5D). For example, IL-4 is a multifunctional cytokine critically involved in inflammation by promoting alternative macrophage activation [13,45,46,47]. Our results show that IL-4 is the foremost upregulated cytokine in myotubes after 48 h rHsGal-1 treatment.

To confirm the cytokine secretome results, we tested cytokine expression in tissue lysates from Bla/J mice treated with PBS or rHsGal-1 for one month. We found a significant increase in IL-4, MCP-1, and TIMP-2 in mice treated with rHsGal-1 compared to the PBS by 38.5, 1.9, and 1.4-fold, respectively (Figure 5E–H). These results may explain the beneficial effect of rHsGal-1 treatment in inflammation and membrane repair.

### 3.6. rHsGal-1 Improves Membrane Repair in Dysferlin Deficient, Patient-Derived Myotubes

**Figure 6:**
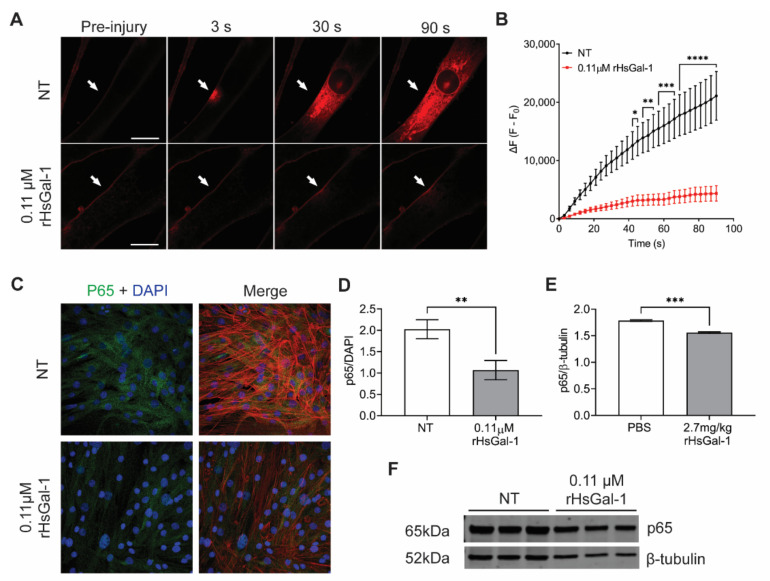
rHsGal-1 treatment modulates membrane repair and inflammation in patient-derived, dysferlin deficient cells. (**A**) Representative images of laser injury assay performed on patient-derived, dysferlin deficient cells. Images were taken at 0, 30, 60, and 90 s post-injury. (**B**) Quantification of laser injury on patient-derived, dysferlin deficient cells. Cells were treated with either 0.11 μM rHsGal-1 or PBS (NT). (**C**) Representative images of dysferlin deficient human myotubes cultured and immunostained with p65 (green), Phalloidin (red), and DAPI (blue). (**D**) Quantification of immunofluorescence of p65 relative to DAPI control in dysferlin deficient human myotubes treated with either 0.11 μM rHsGal-1 or NT. (**E**) Quantification of Western blot for p65 β-tubulin control done on dysferlin deficient human myotubes NT or treated with 0.11 μM rHsGal-1. (**F**) Western blot image for p65 and β-tubulin from dysferlin deficient human myotubes. * *p* < 0.05, ** *p* < 0.01, *** *p* < 0.001, **** *p* < 0.0001 NT vs. rHsGal-1. Bars represent SEM.

With the goal to bring Gal-1 treatment to patients, we used dysferlin-deficient patient-derived myotubes to verify that the therapeutic effects measured in mouse models would translate to a human model. Patient myotubes treated with 0.11 μM rHsGal-1 experienced a 79% reduction in final fluorescent intensity in laser injury assay, indicating increased repair (Figure 6A,B). Changes in inflammatory protein markers were investigated using immunofluorescence and western blots. Quantification of p65 confocal immunofluorescent images revealed a 47% reduction in p65 expression with the same trend using western blot analysis. (Figure 6C–F).

There is a direct relationship between mouse muscle health with cage exploration and rearing [41]. Both of these indices were significantly improved in mice treated with rHsGal-1. We suspect this increase in movement is due to the ability of rHsGal-1 to reduce inflammation and promote muscle membrane repair. Mechanically, we believe the rHsGal-1 is working to facilitate the formation of the membrane patch, an integral component in membrane repair. Together, our results from the one-week and one-month experiments demonstrate that rHsGal-1 affects muscle membrane mechanically through Gal-1 localization to the membrane and biologically through inflammatory signaling [31]. More investigation is needed to show the proteins that rHsGal-1 interacts with as it mediates membrane repair. Regardless of the mechanism, the treatment with rHsGal-1 in BLA/J mice suggests an improvement in muscle health as evidenced in the laser injury, CLAMS cages, biochemical, and histological assays.

Furthermore, rHsGal-1 showed the same therapeutic potential when administered to patient-derived dysferlin-deficient cells (Figure 6). rHsGal-1 shows promise at diminishing the symptoms of LGMD2B in two areas of pathology: inflammation and muscle membrane repair. This two-pronged mechanism would be extremely useful as a therapeutic and may stem from the ability of rHsGal-1 to function as either a monomer or a dimer. The dimer form of rHsGal-1 is clearly more beneficial in assisting in the membrane repair process, while the monomeric version helps to reduce the markers of inflammation. Although more testing is required, these two parallel processes position rHsGal-1 as a highly effective therapeutic against LGMD2B.

## 4. Discussion

Mutations of the dysferlin gene lead to impaired sarcolemma repair with limited treatment options [19,20]. In addition to defective membrane repair, muscles that lack functional dysferlin exhibit chronic muscle inflammation and abnormal lipid metabolism [41,48,49,50]. Steroid, stem cell, or gene replacement therapies to restore the functionality of dysferlin are under investigation [22,51,52]. However, these treatments are still far from clinical application. Our previous study showed that in vitro and ex vivo rHsGal-1 treatment improves myogenesis and membrane repair in dysferlin-deficient models [31]. However, the mechanism behind this improvement is not clear. The multiplicative roles of Gal-1 align with our current results. Here, we provide evidence that rHsGal-1 acts via two discrete mechanisms: restoration of membrane integrity and decrease of inflammatory response in LGMD2B models.

We present data uncovering the biological activity of different types of Gal-1 in membrane repair and inflammation by testing multiple forms of Gal-1 in various oxidation states. An effective therapeutic for LGMD2B patients should address both muscle repair and chronic inflammation in order to reverse pathophysiology. Both rHsGal-1 and reduced dGal-1 effectively improved membrane repair in vitro and ex vivo (Figure 1E–G). Because monomeric and oxidized dGal-1 treatments were ineffective, the reduced dimeric form of Gal-1 is likely responsible for the bulk enhancements in membrane repair in the rHsGal-1 treatment. This aligns with previous studies, which state that the CRD domain of Gal-1 is inactivated via oxidation and that the CRD is necessary for membrane repair [27,31]. The fact that rHsGal-1 and oxidized forms of mGal-1 and dGal-1 both reduced levels of p65 illustrates different niches for the various forms of Gal-1, with oxidized Gal-1 playing a larger role in inflammatory signaling. The dynamic nature of rHsGal-1 allows it to excel at both signaling and membrane repair.

We posit that micro-cellular environmental changes of the various monomer/dimer and redox states of rHsGal-1 are responsible for decreased LGMD2B manifestations. Because of the monomer/dimer modulation, we infer that Gal-1 assists in membrane repair processes as a dimer and simultaneously decreases inflammation as a monomer in our A/J^−/−^, Bla/J, and patient dysferlin-deficient models. Further studies on rHsGal-1 cellular surface and intracellular interactome are needed to deduce additional information on the pathways and mechanisms by which Gal-1 decreases inflammation.

Abnormal expression in the NF-κB pathway causes detrimental effects that accompany inflammation [53]. In accordance with previous studies, we gathered indirect evidence that Gal-1 is associated with downregulated NF-κB activity [31]. For example, phosphorylated p65, which is required for NF-κB relocation to the nucleus, was significantly downregulated in vitro and in vivo [51]. While encouraging, reductions in P-p65 and other elements of the NF-κB pathway do not always correlate with changes in nuclear NF-κB activity or changes in genes being transcribed. However, based on the data we have, we conclude that NF-κB pathway activation is likely also downregulated, although more research, including promotor studies, is needed to confirm this directly. The NF-κB pathway is activated by two different signaling cascades, the canonical and the non-canonical pathways, each with unique signaling and biological functions [54,55]. This study demonstrates that rHsGal-1 is affecting the NF-κB response through the canonical pathway, TAK-1, or the receptor for TAK-1. Since dysregulation of the non-canonical pathway is associated with lymphoid malignancies and autoimmune diseases, it is beneficial that rHsGal-1 is not eliciting this response.

NF-κB activation triggers gene expression for a broad range of pro-inflammatory cytokines, chemokines, and adhesion molecules; therefore, it is unsurprising that Gal-1 treatment affects all of these. The cytokines significantly upregulated with treatment (IL-4, CXCL1, MCP1, and TIMP-2) play a unique role in the cell and may further explain the therapeutic effect of exogenous Gal-1 treatment. IL-4 is a well-studied anti-inflammatory cytokine involved in myogenesis [13,46,47] and tissue repair. In a recently published study, direct treatment with IL-4 improved muscle differentiation [13,56]. This is interesting since Gal-1 treatment in mouse and human models of both Duchenne and LGMD2B both increase muscle differentiation. It should be noted that many cytokines and chemokines have alternative promotor elements that may have consequences unrelated to the NF-κB pathway, which require further investigation. Thus, it is reasonable to conclude that Gal-1 upregulation of Il-4 might drive these changes in differentiation.

Additionally, IL-4 is involved in changing the polarization of macrophages from M1 to M2 [57,58,59]. M1 macrophages are upregulated in dysferlin deficient muscle, which has been shown to contribute to muscle damage [60]. It is possible that rHsGal-1 might polarize macrophages in Bla/J mice through the upregulation of IL-4. This signaling may be the avenue that ultimately leads to greater muscle health. Therefore, upregulation of IL-4 in response to rHsGal-1 treatment may reduce the negative effects of chronic inflammation in LGMD2B and lead to greater muscle health (Figure 5). The CXCL1, MCP-1, and TIMP-2 cytokines are involved in tissue regeneration, wound healing, and ECM regulation, respectively, all of which can contribute to overall muscle health in vivo [61,62,63]. We suspect that these cytokines contribute toward the therapeutic action of Gal-1, but more investigation is necessary.

Previous research provides evidence that activation of NF-κB can increase Gal-1 transcription [64]. Thus, seeing an increase in Gal-1 transcript would be expected when NF-κB is activated. Gal-1 is upregulated in diseases with chronic injuries, such as other types of muscular dystrophy [12]. Specifically, in LGMD2B, Gal-1 transcript levels are 3.8 times higher in patient tissue over non-diseased tissue [12]. Although the level of endogenous Gal-1 in Bla/J mice is unknown, WT and diseased cell models showed similar transcript levels of Gal-1, as shown in our previous study. However, the mRNA levels were upregulated 2-fold with the Gal-1 treatment [31]. Even though the impact of adding exogenous Gal-1 in WT mice has not been defined, studies have shown that IP injection of Gal-1 to treat inflammation in other diseased tissues leads to upregulated endogenous Gal-1 over a period of 24 h [65]. Our results show that, along with negative NF-κB modulation, there is an increase in endogenous Gal-1 transcription due to our exogenous rHsGal-1 treatment compared to PBS treated Bla/J mice. These data suggest a novel self-upregulation loop where Gal-1 positively modulates its own expression. This positive feedback loop could be related to the role of Gal-1 as an alternative pre-mRNA splicing factor, other unknown nuclear interactions, or a yet-to-be-defined signaling pathway [24]. This phenomenon deserves further consideration and experiments to clarify the mechanism of how Gal-1 is able to self-upregulate.

The results obtained during our one-week dose-response experiment show that there is a 6-fold therapeutic range at which rHsGal-1 was effective. The effective concentration seems to peak at 2.7 mg/kg rHsGal-1 every seven days. Although the following doses did not provide benefit: 0.27 mg/kg D0, D7; 0.54 mg/kg D0, D7; and 13.5 mg/kg D0, D7, they also did not result in worsening membrane repair. This shows a safety profile of doses between 0.54 and 13.5 mg/kg in Bla/J mice. This 25-fold safe dosing range, along with pharmacokinetic studies, shows that the treatment of rHsGal-1 takes approximately 12 h to return to pre-dosing levels of Gal-1, indicating that this biologic may be a safe option for human patients.

Functional, histological, and biochemical experiments in our one-month study provide additional evidence of therapeutic benefits with rHsGal-1 treatment. Decreased inflammation may be a fundamental reason for observed increased muscle integrity in treated animals, as inflammation has been shown to play a large role in the pathological symptoms of LGMD2B [12,66]. A primary histological marker for LGMD2B is lipid deposition in affected muscles. This fatty infiltration has been linked to the presence of inflammatory markers [66]. Decreased perilipin with rHsGal-1 treatment demonstrates a decrease in fat deposition within Bla/J myofibers. Biologically, rHsGal-1 decreases inflammation through the NF-κB pathway and upregulates cytokines with anti-inflammatory and regenerative effects. We hypothesize that the decrease in fat deposition is due to decreased activation of the canonical NF-κB pathway as a result of treatment.

There is a direct relationship between mouse muscle health with cage exploration and rearing [41]. Both of these indices were significantly improved in mice treated with rHsGal-1. We suspect that this increase in movement is due to the ability of rHsGal-1 to reduce inflammation and promote muscle membrane repair. We believe the rHsGal-1 works mechanically to facilitate the formation of the membrane patch, an integral component in membrane repair. Together, our results from the one-week and one-month experiments demonstrate that rHsGal-1 affects the muscle membrane mechanically by Gal-1 localization to the membrane and biologically through inflammatory signaling [31]. More investigation is needed to show the proteins that rHsGal-1 interacts with as it mediates membrane repair. Regardless of the mechanism, the treatment with rHsGal-1 in Bla/J mice suggests an improvement in muscle health as evidenced by the CLAMS cages, the laser injury, biochemical, and histological assays.

Furthermore, rHsGal-1 showed the same therapeutic potential when administered to patient-derived dysferlin-deficient cells (Figure 6). rHsGal-1 shows promise at diminishing the symptoms of LGMD2B in two areas of pathology: inflammation and muscle membrane repair. This two-pronged mechanism would be extremely useful as a therapeutic and may stem from the ability of rHsGal-1 to function as either a monomer or a dimer. The dimer form of rHsGal-1 is clearly more beneficial in assisting in the membrane repair process, while the monomeric version helps to reduce the markers of inflammation. Although more testing is required, these two parallel processes position rHsGal-1 as a highly effective therapeutic against LGMD2B.

## 5. Patents

The University of Nevada-Reno has been issued a patent in the U.S. (# US20130065242 A1) and Australia (# 45557BOA/VPB) for “Methods for diagnosing, prognosing and treating muscular dystrophy”. PMVR is an inventor on these patents. Strykagen currently holds the license for this technology.

Brigham Young University has a patent for “Galectin-1 immunomodulation and myogenic improvements in muscle diseases and autoimmune disorders.” (#U.S. Pat. No. 62/161,027. PCT/US2021/026232). PMVR and MLVZ are the inventors of this patent. This does not alter our adherence to MDPI-Cells policies on sharing data and materials.

## Figures and Tables

**Table 1:** Membrane repair improvement factor based on the treatment dosage and schedule. (*p*-values. * *p* < 0.05, ** *p* < 0.01, *** *p* < 0.001, **** *p* < 0.0001).

rHsGal-1 Dose	Treatment Schedule	Fold Improvement Over PBS
0.27 mg/kg	Day 0, 7	1.29 (ns)
0.27 mg/kg	Day 0, 2, 4, 6	1.64 (**)
0.54 mg/kg	Day 0, 7	1.08 (ns)
1.35 mg/kg	Day 0, 7	2.75 (****)
2.7 mg/kg	Day 0, 7	3.35 (****)
2.7 mg/kg	Day 0, 5	1.56 (***)
2.7 mg/kg	Day 0	1.52 (**)
2.7 mg/kg	Day 7	1.99 (***)
5.4 mg/kg	Day 0, 7	2.39 (****)
8.1 mg/kg	Day 0, 7	1.56 (*)
13.5 mg/kg	Day 0, 7	0.98 (ns)
27 mg/kg	Day 0, 3, 7	0.55 (**)

## Data Availability

Data from this research is available upon request to the corresponding author.

## Supplementary Materials

The following are available online at https://www.mdpi.com/article/10.3390/cells10113210/s1, Figure S1: Structure and Purification of different types of Gal-1. Figure S2: In vitro treatment with rHsGal-1 treatment decreases expression of the phosphorylated p65. Figure S3: rHsGal-1 Dose optimization in explant and in vivo Bla/J myofibers. Figure S4: 2.7 mg/kg is the best dose of rHsGal-1 improving membrane repair in explant and in vivo Bla/J myofibers. Figure S5: Quantification of the percentage of twitching myotubes. ****p < 0.0001 between NT and rHsGal-1 treated myotubes. Video S1: Effect of rHsGal-1 on twitching ability of A/J^−/−^ myotubes.
