# Supplementary material for: Therapeutic Benefit of Galectin-1: Beyond Membrane Repair, a Multifaceted Approach to LGMD2B
Source: Cells. 2021 Nov 17;10(11):3210. doi: 10.3390/cells10113210 (PMC8621416; doi:10.3390/cells10113210)
Supplement: Supplementary file 1 [file cells-10-03210-s001.zip › supplementary/LEGENDS- Supplemental Figures.pdf]

Supplementary Figures and Video

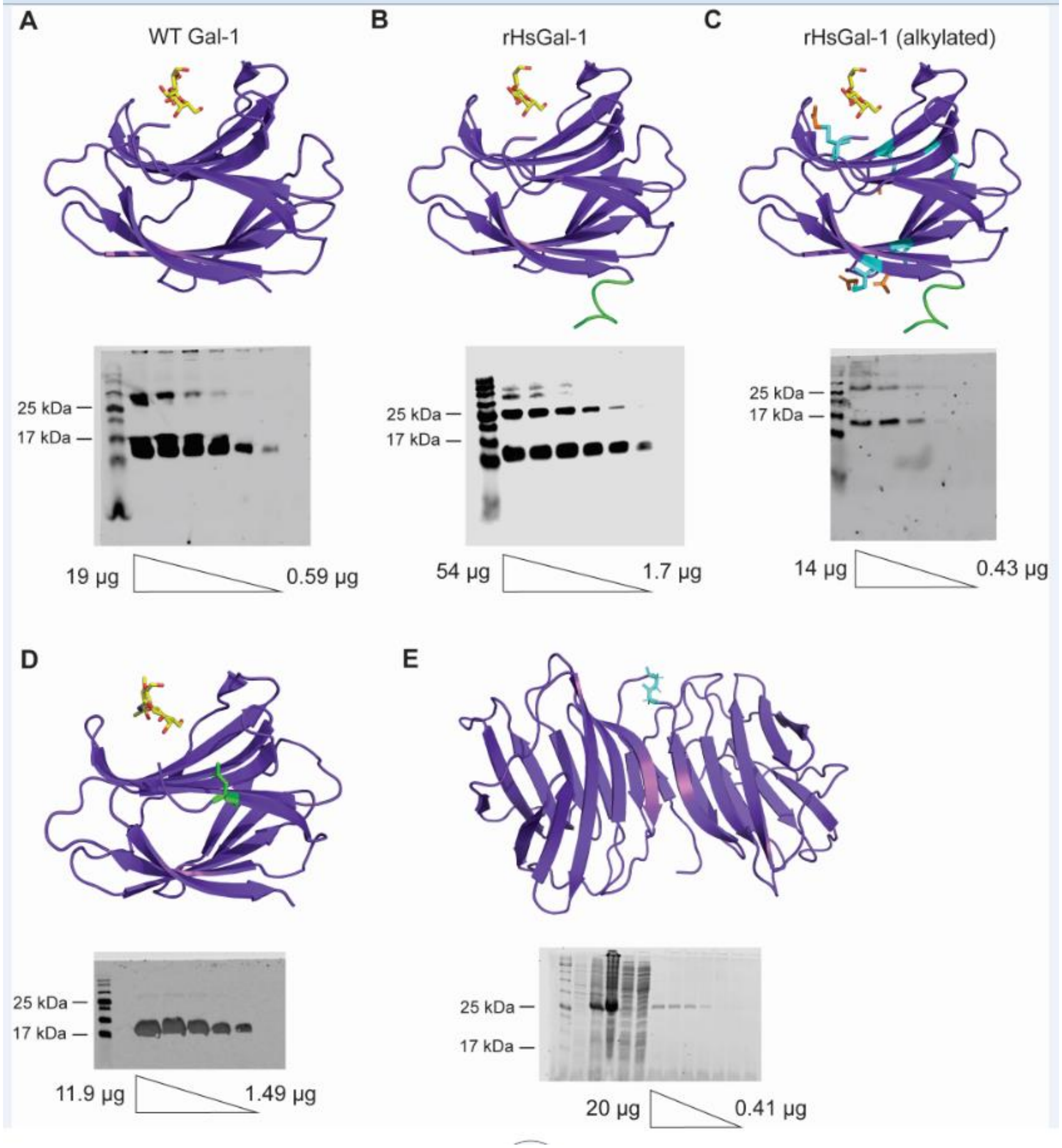

Figure S1. Structure and Purification of different types of Gal-1. **(A)** Crystal structure of wild-type galectin-1 (WT Gal-1) bound to generic β-galactoside ligand (PDB 1GZW) and western blot image of WT Gal-1at decreased dosage. **(B).** Model of recombinant human galectin-1 (rHsGal-1) bound to generic β-galactoside ligand (modeled from PDB 1GZW) and western blot image of rHsGal-1 at decreased dosage. **(C)** Model of alkylated rHsGal-1 bound to generic β-galactoside ligand (modeled from PDB 1GZW) and western blot image of alkylated rHsGal-1 at decreased dosage. **(D)** Representative structure of the fixed monomeric form of Gal-1 and western blot image

with serial dilution for verification of proper construction. **(E)** Representative structure of the fixed dimeric form of Gal-1 and western blot image with serial dilution for verification of proper construction.

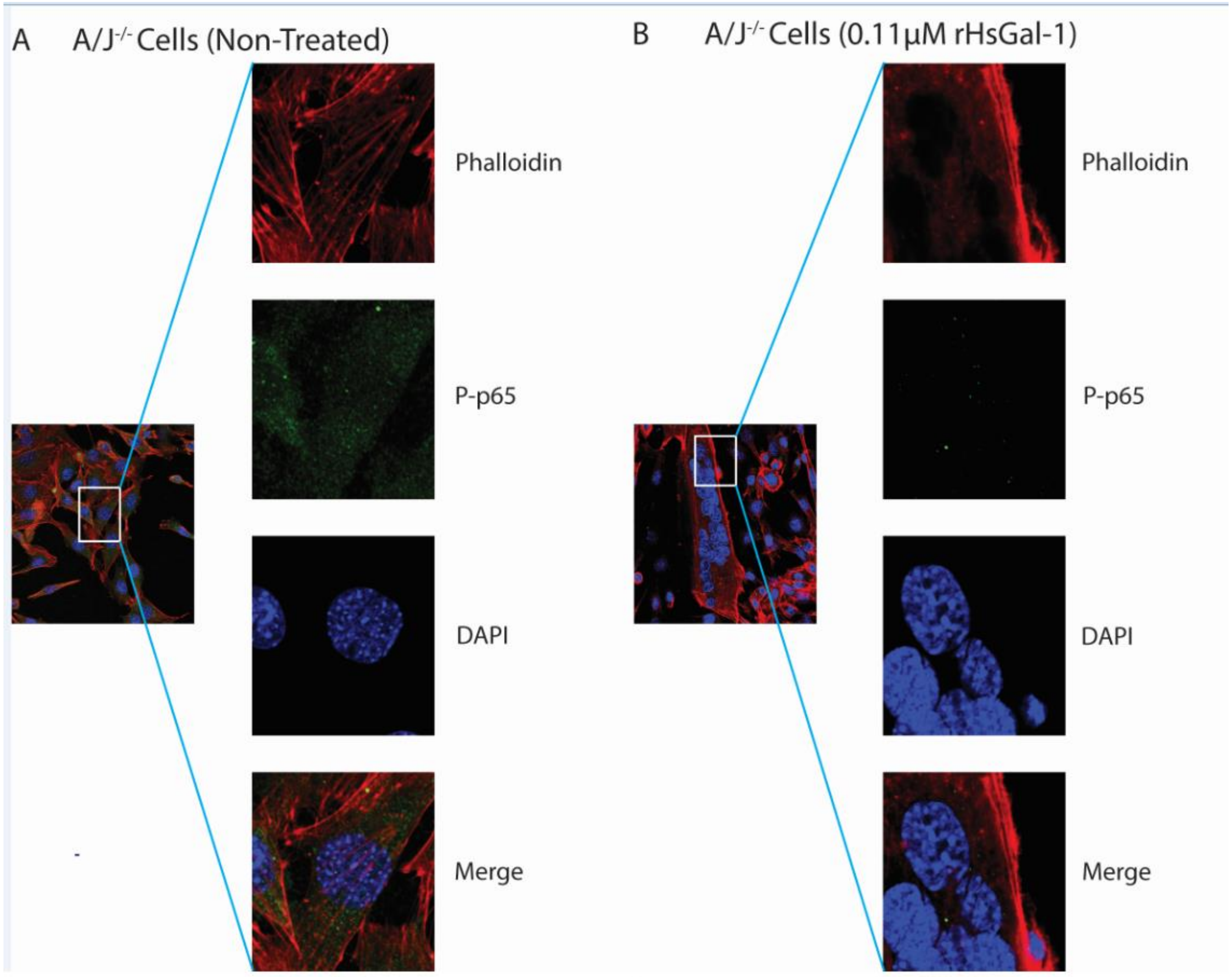

Figure S2. In vitro treatment with rHsGal-1 treatment decreases expression of the phosphorylated p65. Representative images of NT (A) or 48 h treated A/J$^{-/-}$ myotubes with 0.11µM rHsGal-1 in (B). A/J$^{-/-}$ NT or 0.11µM rHsGal-1 treated myotubes cultured and immunostained with Phospho-p65 (green), Phalloidin (red), and DAPI (blue).

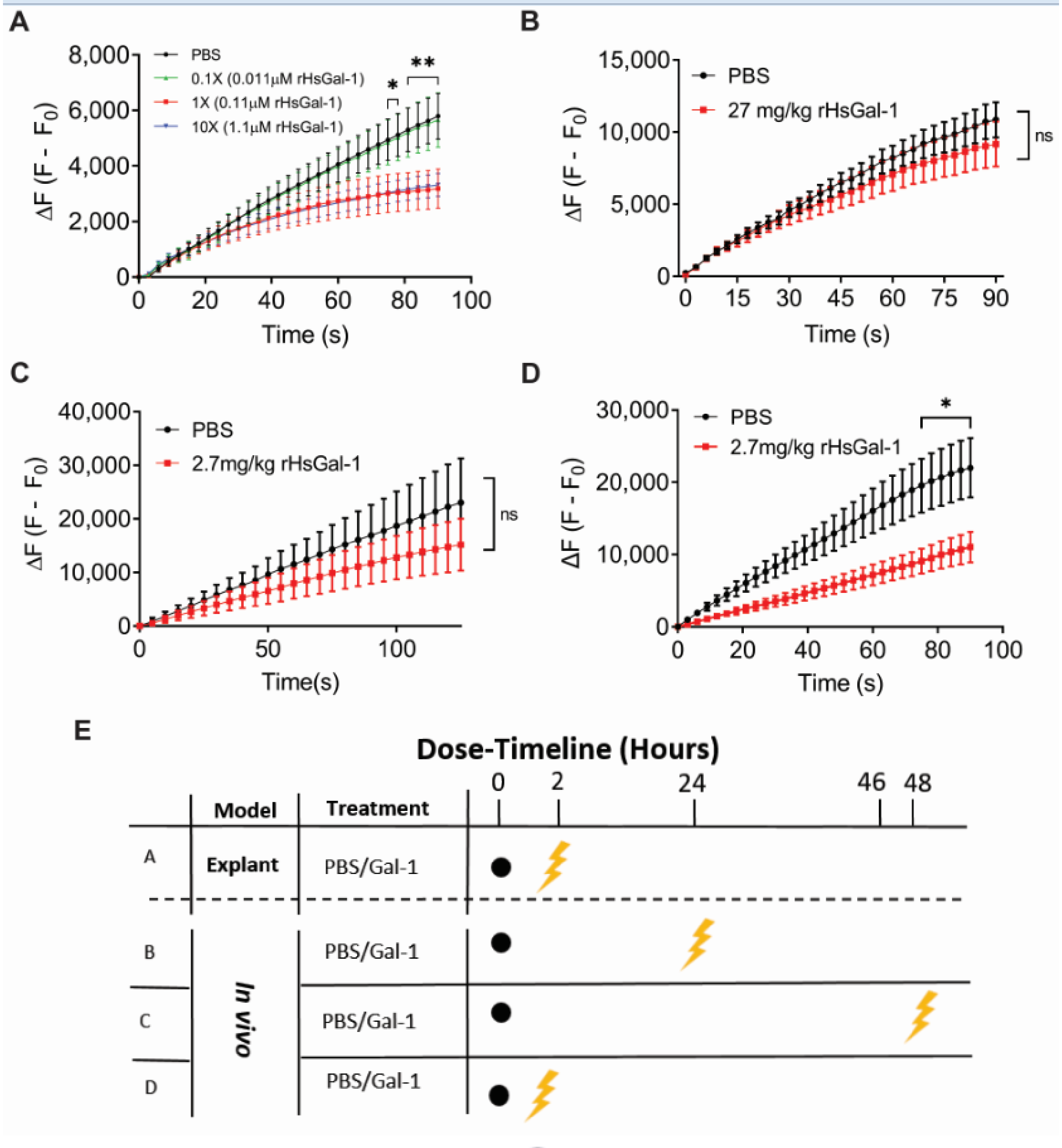

Figure S3. rHsGal-1 Dose optimization in *explant* and in vivo Bla/J myofibers. **(A)** Quantified laser injury assay on explant mouse muscles from BLA/J mice treated with 0.011 µM rHsGal-1 (0.1X), 0.11 µM rHsGal-1 (1X), 1.1 µM rHsGal-1 (10X) compared to PBS treated control. **(B)** Quantified laser injury assay on mouse muscles from BLA/J mice treated in vivo 27 mg/kg rHsGal-1 (10X) compared to PBS treated control. Muscles were taken and injured 24 h after treatment. **(C)** Quantified laser injury assay on mouse muscles from BLA/J mice treated in vivo 2.7 mg/kg rHsGal-1 (1X) compared to PBS treated control. Muscles were taken and injured 48 h after treatment. **(D)** Quantified laser injury assay on mouse muscles from BLA/J mice treated in vivo 2.7 mg/kg rHsGal-1 (1X) compared to PBS treated control. Muscles were taken and injured 2 h

after treatment. **(E)** Timeline for experiments shown in A-E. Black dot = treatment. Yellow bolt = injury. Values were measured by Tukey's multiple comparison test and indicated by: ****p < 0.0001, ***p < 0.001, **p < 0.01, and *p < 0.05 between control and rHsGal-1 treated mice.

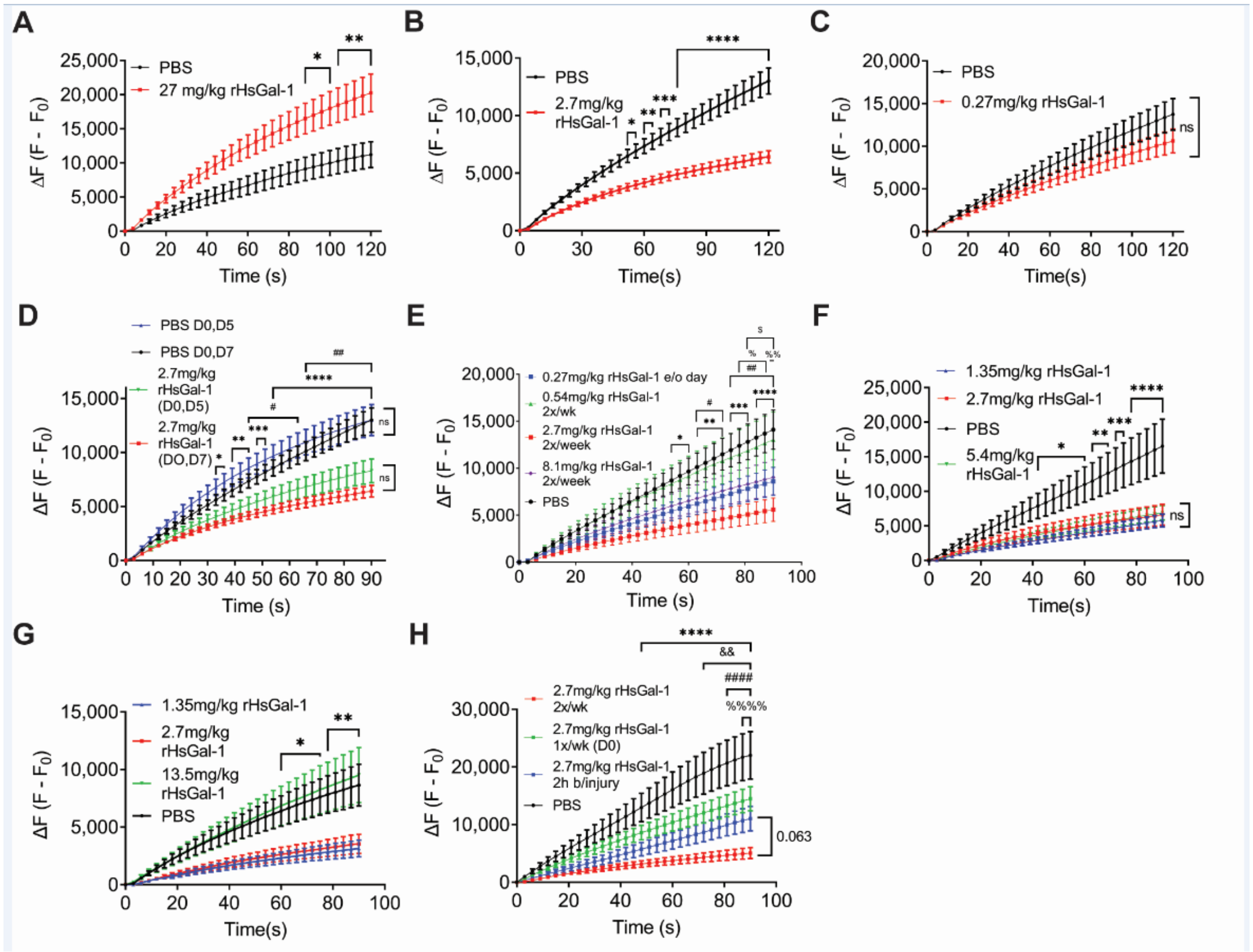

Figure S4. 2.7 mg/kg is the best dose of rHsGal-1 improving membrane repair in explant and in vivo Bla/J myofibers. **(A)** Quantified laser injury assay on BLA/J mouse muscles treated in vivo with 27 mg/kg rHsGal-1 compared to PBS treated control. Mice were treated three times in 7 days, and muscles were taken on the seventh day to be injured and imaged. **(B)** Quantified laser injury assay on mouse muscles from BLA/J mice treated in vivo 2.7 mg/kg rHsGal-1 compared to PBS treated control. Mice were treated on day 0 and day 7. Muscles were taken and injured 7 days after first treatment. **(C)** Quantified laser injury assay on mouse muscles from BLA/J mice treated in vivo 0.27 mg/kg rHsGal-1 compared to PBS treated control. Mice were treated on day 0 and day 7. Muscles were taken and injured 7 days after first treatment. **(D)** Quantified laser injury assay on mouse muscles from BLA/J mice treated in vivo 2.7 mg/kg rHsGal-1 (1X) compared to PBS treated control. One rHsGal-1 and one PBS-treated mouse were treated on days 0 and 7, while the other rHsGal-1 and PBS-treated mice were treated on days 0 and 5. Muscles were taken and injured 7 days after first treatment. * = PBS vs. 2.7 mg/kg rHsGal-1 (D0, D7); # = PBS vs. 2.7 mg/kg rHsGal-1 (D0, D5). **(E)** Quantified laser injury assay on mouse muscles from BLA/J mice treated in vivo with 0.27 mg/kg rHsGal-1 every other day, 0.54 mg/kg rHsGal-1 on days 0 and 7, 2.7 mg/kg rHsGal-1 on days 0 and 7, and 8.1 mg/kg rHsGal-1 on days 0 and 7 compared to PBS treated control. All muscles were taken and injured 7 days after first treatment. # = 0.54 mg/kg vs.

2.7mg/kg; * = PBS vs. 2.7 mg/kg; % = PBS vs. 0.27 mg/kg; $ = PBS vs. 8.1 mg/kg. **(F)** Quantified laser injury assay on mouse muscles from BLA/J mice treated in vivo with 1.35 mg/kg rHsGal-1, 2.7 mg/kg rHsGal-1, and 5.4 mg/kg rHsGal-1 compared to PBS treated control. Mice were treated on days 0 and 7. Muscles were taken 7 days after first treatment. **(G)** Quantified laser injury assay on mouse muscles from BLA/J mice treated in vivo with 1.35 mg/kg rHsGal-1, 2.7 mg/kg rHsGal-1, and 13.5 mg/kg rHsGal-1. Mice were treated on days 0 and 7. Muscles were taken 7 days after first treatment. **(H)** Quantified laser injury assay on mouse muscles from BLA/J mice treated in vivo with 2.7 mg/kg rHsGal-1 or PBS control. Mice were treated on days 0 and 7, day 0 only, or day 7 only. * = PBS vs. rHsGal-1 D0,7; # = PBS vs. rHsGal-1 D7 only; %= rHsGal-1 D0,7 vs. rHsGal-1 D0 only; and = PBS vs. rHsGal-1 D0 only. Values for all graphs were measured by Tukey's multiple comparison test and indicated except where noted by: ****$p < 0.0001$, ***$p < 0.001$, **$p < 0.01$, and *$p < 0.05$ between control and rHsGal-1 treated mice.

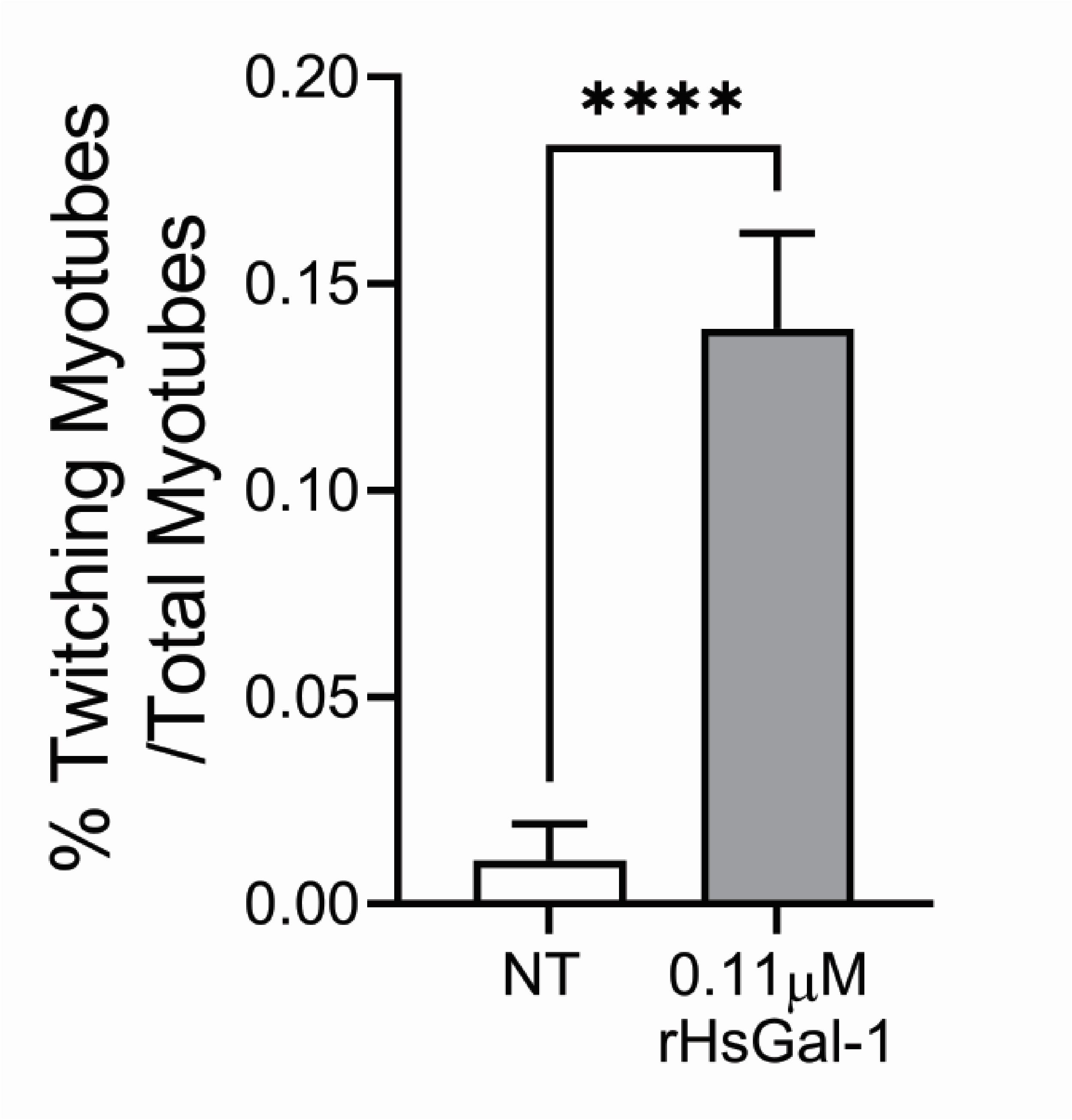

Figure S5. Quantification of the percentage of twitching myotubes. ****$p < 0.0001$ between NT and rHsGal-1 treated myotubes.

Supplemental Video 1. Effect of rHsGal-1 on twitching ability of A/J$^{-/-}$ myotubes. Video taken 15 days post-treatment, comparing T and NT myotubes.
